# Supplementary material for: Benchmarking AutoML frameworks for disease prediction using medical claims
Source: BioData Min. 2022 Jul 26;15:15. doi: 10.1186/s13040-022-00300-2 (PMC9327416; doi:10.1186/s13040-022-00300-2)
Supplement: Supplementary file 1 — Additional file 1 ROC AUC performance of different AutoML models trained for various disease outcomes from non-stratified bootstrap samples. Median values are indicated by diamond markers and 95% CI limits are indicated by circles. [file 13040_2022_300_MOESM1_ESM.pdf]

SHORT REPORT ADDITIONAL FILE

Additional File for Benchmarking AutoML Frameworks for Disease Prediction Using Medical Claims

Full list of author information is available at the end of the article

Figures

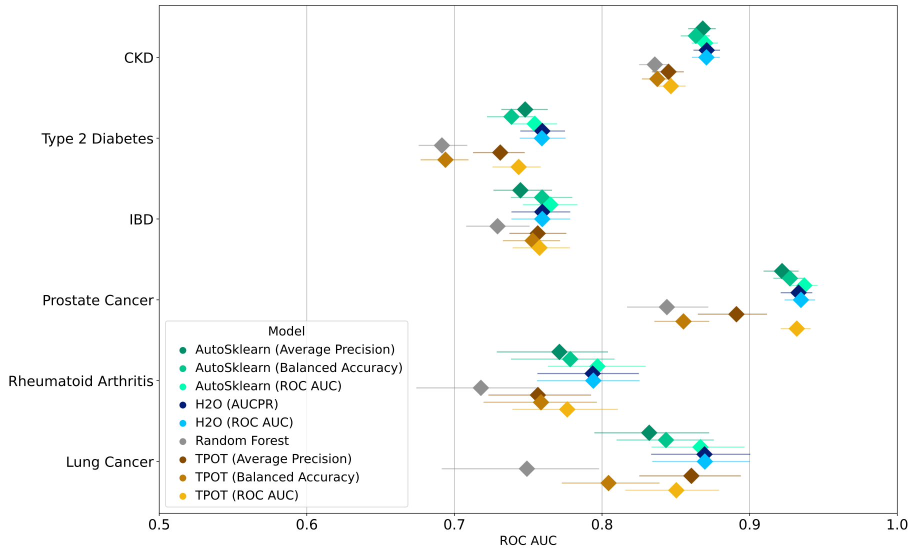

Figure 1 ROC AUC performance of different AutoML models trained for various disease outcomes from non-stratified bootstrap samples. Median values are indicated by diamond markers and 95% CIs are indicated by lines.

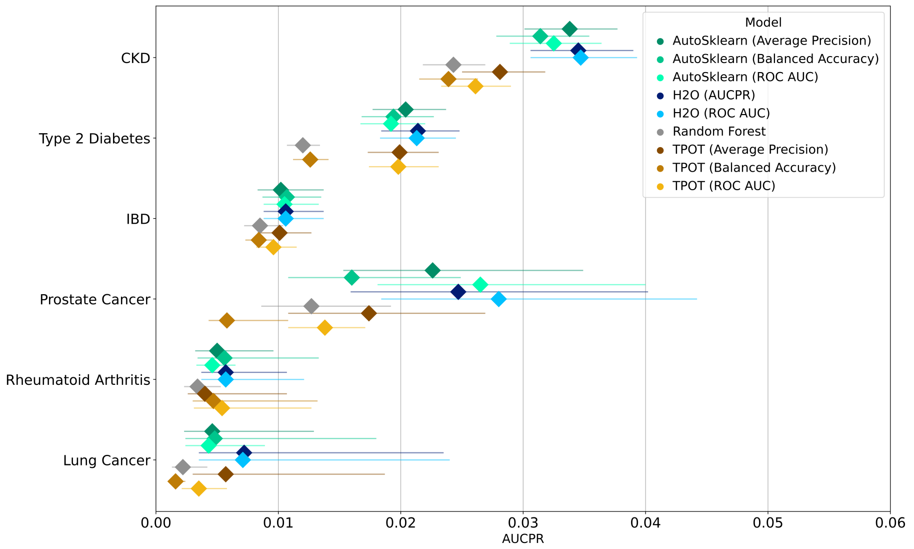

Figure 2 AUCPR performance of different AutoML models trained for various disease outcomes from non-stratified bootstrap samples. Median values are indicated by diamond markers and 95% CIs are indicated by lines.

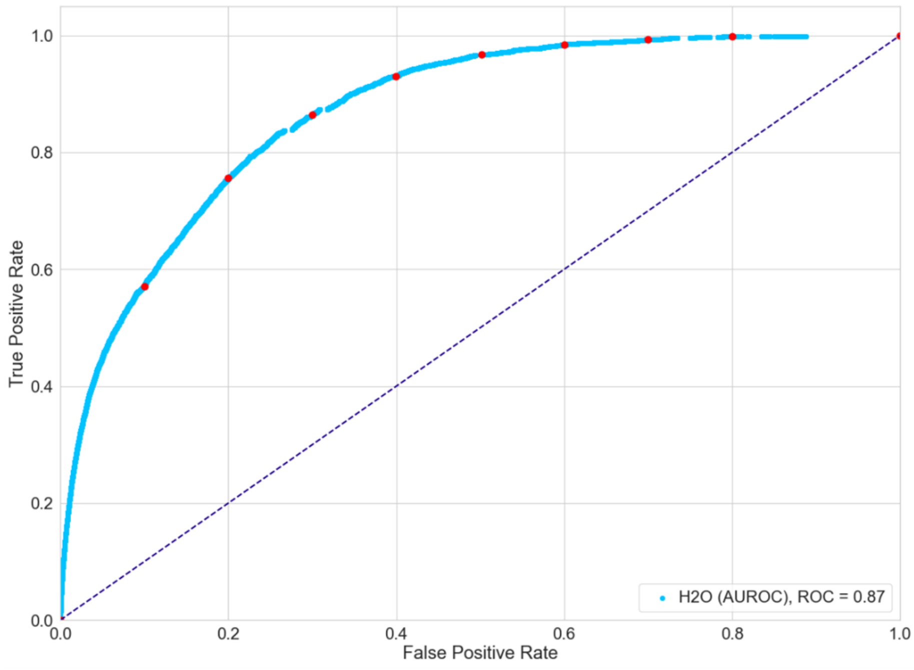

Figure 3 ROC curve of H2O (AUROC) model for predicting lung cancer on validation set. Red dots indicate locations of decile probabilities.

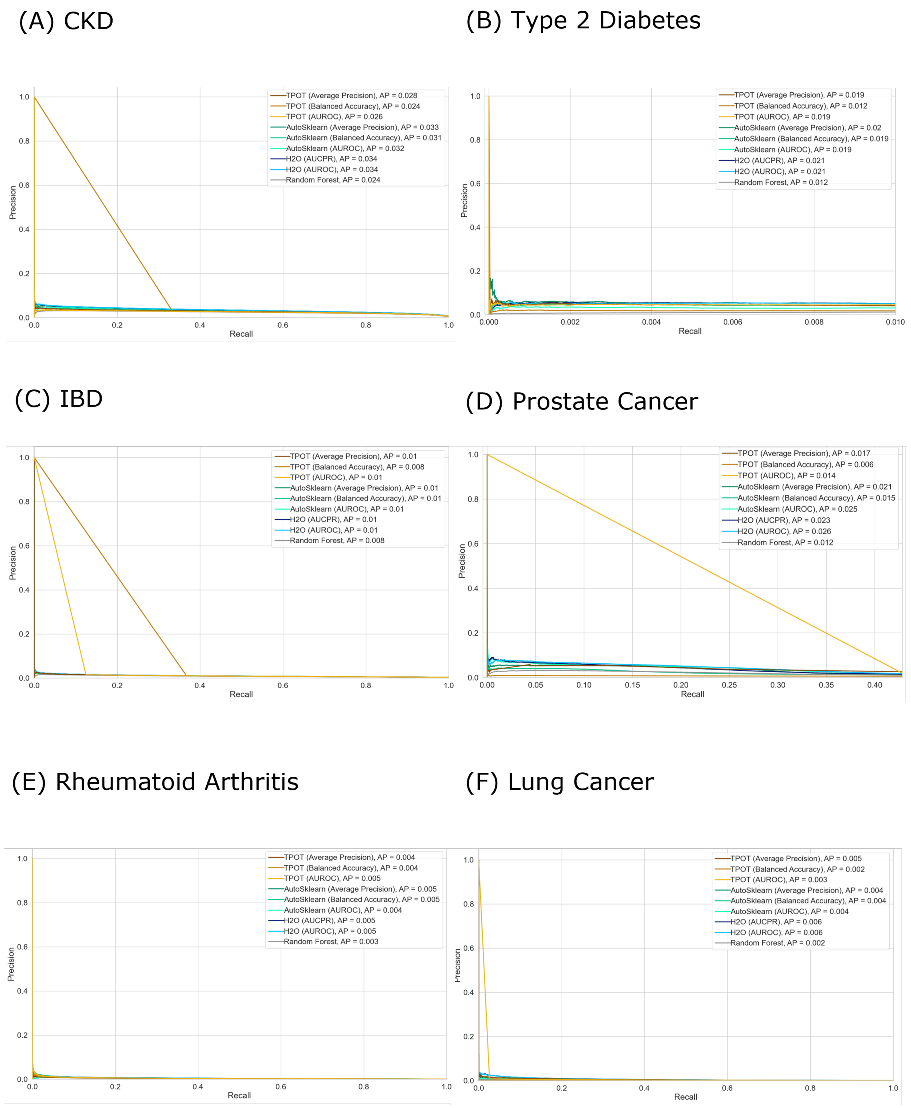

Figure 4 Area under the precision-recall curve (AUCPR) curves of models trained for predicting different diseases showing values from Recall = 0 to Recall = 1.0. AUCPR curves are generated using prediction scores on full validation set (N = 12,125,832).

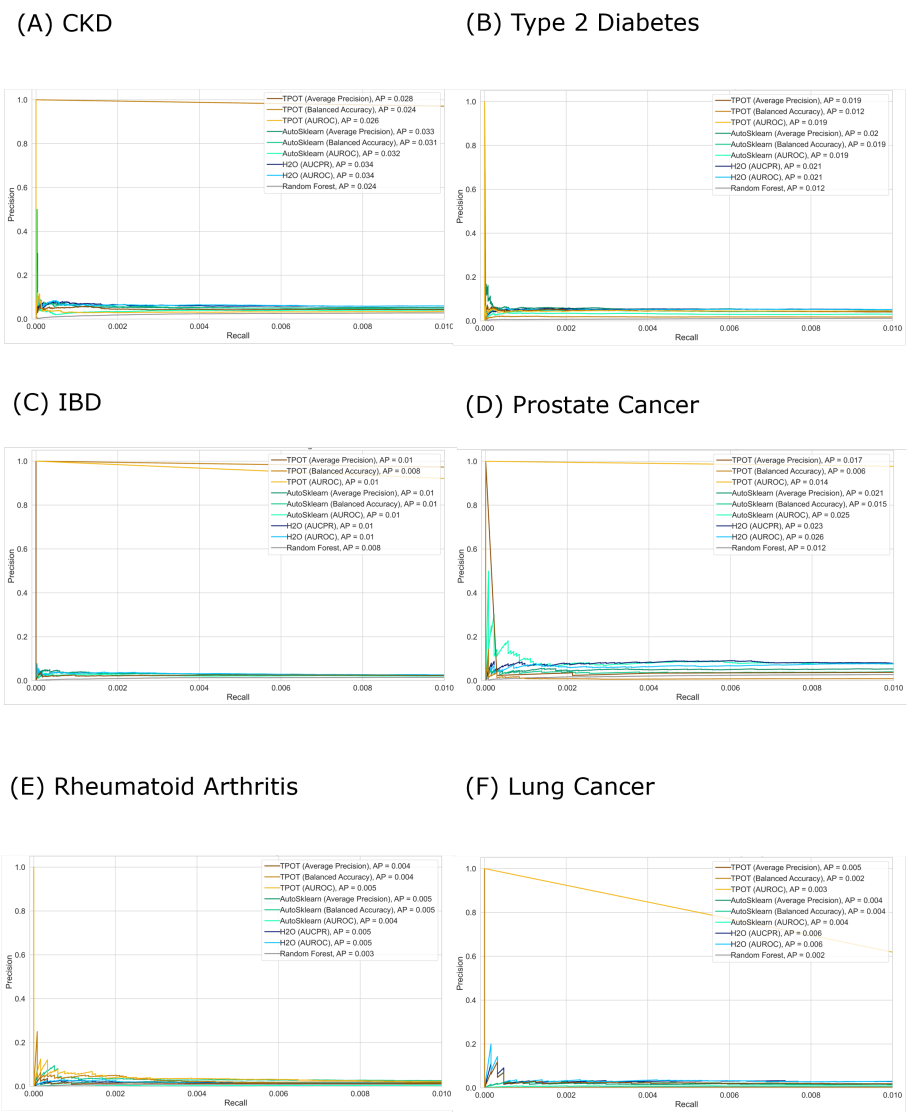

Figure 5 Area under the precision-recall curve (AUCPR) curves of models trained for predicting different diseases showing values from Recall = 0 to Recall = 0.01. AUCPR curves are generated using prediction scores on full validation set (N = 12,125,832).

Tables

	AutoSklearn (Average Precision)	AutoSklearn (Balanced Accuracy)	AutoSklearn (ROC AUC)	H2O (AUC)	H2O (AUCPR)	TPOT (Average Precision)	TPOT (Balanced Accuracy)	TPOT (ROC AUC)	Random Forest
**CKD**	0.8685 (0.8604, 0.876)	0.8638 (0.855, 0.8721)	0.8702 (0.8623, 0.8782)	0.8712 (0.8632, 0.879)	**0.8714 (0.8631, 0.8794)**	0.8455 (0.8347, 0.8555)	0.8374 (0.8279, 0.8475)	0.847 (0.8382, 0.8562)	0.8358 (0.8257, 0.8455)
**Type 2 Diabetes**	0.7466 (0.7307, 0.7623)	0.7382 (0.7212, 0.7526)	0.7538 (0.7385, 0.769)	**0.7585 (0.7425, 0.7725)**	0.7584 (0.743, 0.7728)	0.7295 (0.7112, 0.7462)	0.6926 (0.6752, 0.7087)	0.7425 (0.7252, 0.7582)	0.6907 (0.6753, 0.7064)
**IBD**	0.745 (0.7239, 0.7646)	0.7597 (0.7385, 0.7775)	0.7642 (0.7446, 0.7825)	**0.7593 (0.7399, 0.7773)**	**0.7593 (0.7399, 0.7773)**	0.7563 (0.7381, 0.7746)	0.7522 (0.7341, 0.7702)	0.7578 (0.7373, 0.7763)	0.729 (0.7068, 0.7495)
**Rheumatoid Arthritis**	0.7699 (0.7346, 0.8087)	0.7771 (0.7433, 0.8143)	0.7974 (0.7629, 0.8288)	**0.7952 (0.7588, 0.8275)**	0.7948 (0.7576, 0.8275)	0.7578 (0.7213, 0.7931)	0.7599 (0.7236, 0.7961)	0.7788 (0.7416, 0.8118)	0.7184 (0.6714, 0.7579)
**Prostate Cancer**	0.9213 (0.9088, 0.9323)	0.9266 (0.9166, 0.9366)	**0.9369 (0.9254, 0.9463)**	0.9347 (0.9233, 0.9448)	0.9328 (0.9216, 0.9425)	0.8897 (0.8627, 0.9094)	0.8553 (0.8378, 0.8739)	0.9315 (0.9202, 0.9415)	0.8447 (0.814, 0.8721)
**Lung Cancer**	0.8325 (0.7877, 0.8725)	0.8448 (0.8075, 0.8757)	0.8665 (0.8333, 0.8996)	**0.8712 (0.8366, 0.9067)**	0.8711 (0.8365, 0.9071)	0.8602 (0.8261, 0.8933)	0.8055 (0.7726, 0.8394)	0.8485 (0.8155, 0.886)	0.7519 (0.7003, 0.8124)

Table 1 Median ROC AUC performance (95% CI) of different AutoML models determined from stratified bootstrap samples of predictions on the validation set. Models with the best performance for each disease are indicated in bold.

	AutoSklearn (Average Precision)	AutoSklearn (Balanced Accuracy)	AutoSklearn (ROC AUC)	H2O (AUC)	H2O (AUCPR)	TPOT (Average Precision)	TPOT (Balanced Accuracy)	TPOT (ROC AUC)	Random Forest
**CKD**	0.0339 (0.0309, 0.038)	0.0316 (0.0289, 0.0352)	0.0325 (0.0298, 0.0359)	**0.0347 (0.0317, 0.0384)**	0.0345 (0.0313, 0.0386)	0.0282 (0.0257, 0.0314)	0.0239 (0.0223, 0.0256)	0.0261 (0.0239, 0.029)	0.0243 (0.0223, 0.0264)
**Type 2 Diabetes**	0.0201 (0.0174, 0.0231)	0.0193 (0.0167, 0.0221)	0.0191 (0.0171, 0.0216)	0.021 (0.0185, 0.0244)	**0.0212 (0.0186, 0.0246)**	0.0198 (0.0172, 0.0226)	0.0124 (0.0113, 0.0138)	0.0198 (0.0173, 0.0224)	0.012 (0.0109, 0.013)
**IBD**	0.0103 (0.0086, 0.0131)	0.0107 (0.009, 0.0138)	0.0105 (0.009, 0.013)	**0.0107 (0.009, 0.0135)**	**0.0107 (0.009, 0.0135)**	0.0102 (0.0086, 0.0124)	0.0083 (0.0075, 0.0092)	0.0096 (0.0085, 0.0111)	0.0085 (0.0074, 0.0099)
**Rheumatoid Arthritis**	0.005 (0.0033, 0.0094)	0.0056 (0.0036, 0.0129)	0.0046 (0.0035, 0.0062)	**0.0058 (0.004, 0.0105)**	**0.0058 (0.004, 0.0103)**	0.0039 (0.0027, 0.0087)	0.0045 (0.003, 0.0116)	0.0053 (0.0034, 0.0136)	0.0034 (0.0023, 0.0052)
**Prostate Cancer**	0.0227 (0.0155, 0.0324)	0.016 (0.0114, 0.0242)	0.0267 (0.0181, 0.0396)	**0.0281 (0.0189, 0.0415)**	0.0249 (0.0168, 0.038)	0.0171 (0.0114, 0.025)	0.0058 (0.0047, 0.0107)	0.0138 (0.0113, 0.0165)	0.0127 (0.009, 0.0182)
**Lung Cancer**	0.0045 (0.0024, 0.0122)	0.0047 (0.0025, 0.0149)	0.0043 (0.0026, 0.0084)	0.0074 (0.0038, 0.0216)	**0.0075 (0.0038, 0.0211)**	0.0059 (0.0032, 0.0189)	0.0016 (0.0012, 0.0023)	0.0035 (0.0023, 0.0058)	0.0023 (0.0014, 0.0041)

Table 2 Median AUCPR performance (95% CI) of different AutoML models determined from stratified bootstrap samples of predictions on the validation set. Models with the best performance for each disease are indicated in bold.

Percentile	Threshold	TN	FP	FN	TP	FPR	TPR
10	0.000050	1332	12118119	0	6381	1.000	1.000
20	0.000063	2420542	9698909	13	6368	0.800	0.998
30	0.000076	3637704	8481747	46	6335	0.700	0.993
40	0.000100	4850231	7269220	101	6280	0.600	0.984
50	0.000148	6038573	6080878	211	6170	0.502	0.967
60	0.000247	7275056	4844395	443	5938	0.400	0.931
70	0.000412	8486907	3632544	868	5513	0.300	0.864
80	0.000627	9699105	2420346	1559	4822	0.200	0.756
90	0.001109	10910511	1208940	2737	3644	0.100	0.571
100	0.061339	12119450	1	6381	0	0.000	0.000

Table 3 Confusion matrices for H2O (AUROC) model for predicting lung cancer for thresholds set at decile values of validation set prediction probabilities.
